# Untargeted Metabolomics Reveals Regional and Cultivar-Associated Metabolomic Variation in Soybeans Across Northeast China

**DOI:** 10.3390/ijms27198925

**Published:** 2026-10-08

**Authors:** Jinghui Wang, Lupeng Gou, Haohai You, Feng Wang, Jie Zhu, Yinqi Wang, Zijian Xia, Zhuoduojie Kuan, Zhaohui Wang

**Affiliations:** 1 Institute for the Smart Agriculture, Jilin Agricultural University, Changchun 130118, China; wjh3205@jlau.edu.cn (J.W.); goulupeng@mails.jlau.edu.cn (L.G.); 2 College of Information Technology, Jilin Agricultural University, Changchun 130118, China; 18112847628@163.com (F.W.); 19937722944@163.com (J.Z.); aaaprils11@gmail.com (Z.X.); 15543133299@163.com (Z.K.); 3 School of Information Science and Engineering, Northeastern University, Shenyang 110819, China; youhaohai@mails.neu.edu.cn; 4 College of Food Science and Engineering, Jilin Agricultural University, Changchun 130118, China; wyqi7x@icloud.com

**Keywords:** soybean, non-targeted metabolomics, cultivar–location variation, regional metabolomic variation, isoflavones, lipid metabolism

## Abstract

Soybean (Glycine max) quality and metabolic composition are shaped by complex interactions between genetic background and agro-ecological conditions, making metabolic characterization across major production regions valuable for germplasm evaluation and quality-oriented breeding. In this study, an untargeted liquid chromatography–mass spectrometry (LC-MS) metabolomics approach was applied to compare the mature seed metabolomes of nine representative commercial soybean cultivars sampled from two major production regions in Northeast China: Heilongjiang (HLJ, four cultivars from Harbin) and Liaoning (LN, five cultivars from Shenyang). Because region-specific cultivars were collected from a single site and seed lot per province without direct site-level meteorological or soil measurements, genotype and environment are confounded in this observational design, and the observed differences reflect the combined variation in the sampled cultivar–location cohorts. After preprocessing, 8283 features were putatively annotated (MSI Level 2/3). Multivariate statistical analyses (PCA, PLS-DA, and OPLS-DA) combined with cultivar-grouped cross-validation revealed clear metabolic separation between the sampled HLJ and LN cohorts. A total of 1485 putatively annotated metabolites (VIP > 1.0, subsample-level FDR < 0.05, fold change HLJ/LN > 2.0 or <0.5) were screened as exploratory candidate differential metabolites, with 953 exhibiting higher abundance (FC>2.0) and 532 exhibiting lower abundance (FC<0.5, i.e., higher abundance in LN) in HLJ soybeans relative to LN soybeans. Exploratory KEGG pathway mapping (based on nominal p-values, as no pathway reached FDR-adjusted q<0.05) indicated nominal enrichment in isoflavonoid biosynthesis (p=0.0043, q=0.261) and tryptophan metabolism (p=0.0093, q=0.283); specifically, sampled LN cultivar–location combinations exhibited higher steady-state abundances of isoflavones and their glycosylated derivatives, whereas sampled HLJ soybeans exhibited higher steady-state abundances of specific lipid species (unsaturated fatty acyls, sphingolipids) and tryptophan-derived indoleamines. In the absence of direct environmental measurements, potential links between these steady-state metabolic signatures and regional macroclimatic differences (such as thermal regimes or high-latitude cold stress) are proposed strictly as exploratory, literature-informed hypotheses. Overall, these findings provide preliminary candidate metabolic markers for characterizing the sampled regional soybean germplasm; however, independent validation across additional harvest years, multiple geographic sites, independent seed lots, and broader cultivar panels will be required before these candidate metabolites can be considered validated biomarkers for geographic authentication.

## 1. Introduction

Soybean (*Glycine max* (L.) Merr.) is an annual herbaceous crop of global agricultural importance, valued for its high contents of protein, oil, and diverse secondary metabolites [1,2,3]. In addition to its nutritional value, the soybean metabolome comprises a broad range of flavonoids, lipids, amino acids, and other specialized metabolites that contribute to plant growth, stress responses, and seed quality [4,5]. The composition and abundance of these metabolites can vary substantially in response to genetic background and environmental conditions, making the metabolome a potentially informative molecular signature for investigating differences among soybean production regions [6].

China is one of the major soybean-producing countries, with soybean cultivation distributed across several regions characterized by distinct climatic and ecological conditions [7]. Northeast China, particularly Heilongjiang and Liaoning provinces, represents an important soybean production area with substantial variation in latitude, temperature regimes, and other environmental conditions [8]. Such regional differences, together with differences in soybean genetic background and cultivation conditions, may contribute to variation in metabolite accumulation and consequently affect the nutritional and quality characteristics of soybean seeds [9]. Therefore, characterizing region-associated metabolic variation is important for understanding the biochemical diversity of soybean germplasm and production systems in Northeast China.

A variety of analytical approaches have been explored for evaluating the geographical characteristics and authenticity of agricultural products, including mineral element profiling, stable isotope analysis, fatty acid composition, volatile compound analysis, and transcriptomic approaches [10,11,12,13]. These methods have provided valuable information for geographical discrimination and quality assessment; however, many of them focus on specific classes of chemical constituents or biological responses and therefore may not capture the broader metabolic diversity associated with regional variation [14]. Therefore, a comprehensive, non-targeted characterization of the global metabolome is essential. This approach not only resolves the distinct biochemical signatures associated with different soybean production regions but also clarifies the ecophysiological adaptations of these cultivars to their local habitats.

LC-MS-based non-targeted metabolomics provides a comprehensive strategy for profiling a wide range of small molecules without requiring a predefined list of target metabolites [15], and this approach has been increasingly applied to successfully authenticate geographical origins across various agricultural products, including avocado, black goji, and blueberries [16,17,18,19,20]. However, most studies emphasize biomarker discovery rather than characterizing the broader pathway-level metabolomic variations across different cultivar–location systems. The specific regional and cultivar-associated metabolic differences among soybeans grown in the highly contrasting agro-climatic zones of Northeast China remain largely uncharacterized. Northeast China presents a unique ecological gradient. Specifically, soybean cultivars in Heilongjiang (HLJ) are frequently challenged by high-latitude early-season chilling, whereas those in Liaoning (LN) experience relatively higher average temperatures [21,22]. We hypothesize that soybean cultivar–location combinations sampled from these two contrasting zones exhibit pronounced metabolomic divergence—particularly in phenylpropanoid and lipid pathways—reflecting regional and cultivar-associated metabolic variation across different agro-climatic habitats.

Therefore, this study employed a non-targeted LC-MS metabolomics approach to characterize the metabolic profiles of nine representative commercial soybean cultivars collected from two major production regions in Northeast China: Heilongjiang and Liaoning. By integrating global metabolic profiling with multidimensional statistical modeling and exploratory pathway analysis, we aimed to elucidate the candidate differential metabolites and candidate metabolic pathways distinguishing these sampled cultivar–location groups. Because region-specific cultivars were sampled from a single representative station and seed lot per province, the observed metabolic divergence reflects the combined variation in cultivar genotype and local production environment. Accordingly, this study seeks to provide an exploratory metabolic reference for characterizing regional commercial soybean germplasm in Northeast China and to screen preliminary candidate metabolic markers, establishing a baseline for future multi-year, multi-site, multi-lot, and multi-cultivar validation studies required for robust geographic authentication and quality-oriented breeding.

## 2. Results and Discussion

### 2.1. LC-MS Data Analysis

Raw LC-MS data were processed using Progenesis QI v2.3 for baseline correction, peak detection, alignment, and retention-time correction. Metabolite annotation was performed by matching accurate mass (mass error within ±10 ppm), MS/MS fragmentation patterns, and isotope distributions against the HMDB, LIPID MAPS (v2.3), METLIN, and in-house databases. After data preprocessing and quality filtering, 21,893 ion features were detected across the soybean samples, of which 8283 features were assigned putative metabolite annotations (MSI Level 2/3, as authentic reference standards were not co-injected for Level 1 confirmation). Specifically, 3815 features with experimental MS/MS fragmentation matches (Fragmentation Score > 0) were classified as MSI Level 2 putative annotations, whereas the remaining features matched solely by MS1 accurate mass and isotopic distribution (Fragmentation Score = 0) were categorized as MSI Level 3 tentative formula/class matches. Because broad databases (e.g., HMDB and METLIN) contain exogenous and synthetic compounds that are isobaric to endogenous plant isomers, MSI Level 3 hits with non-plant names were treated strictly as ambiguous elemental formula matches rather than confirmed chemical structures.

**Figure 1:**
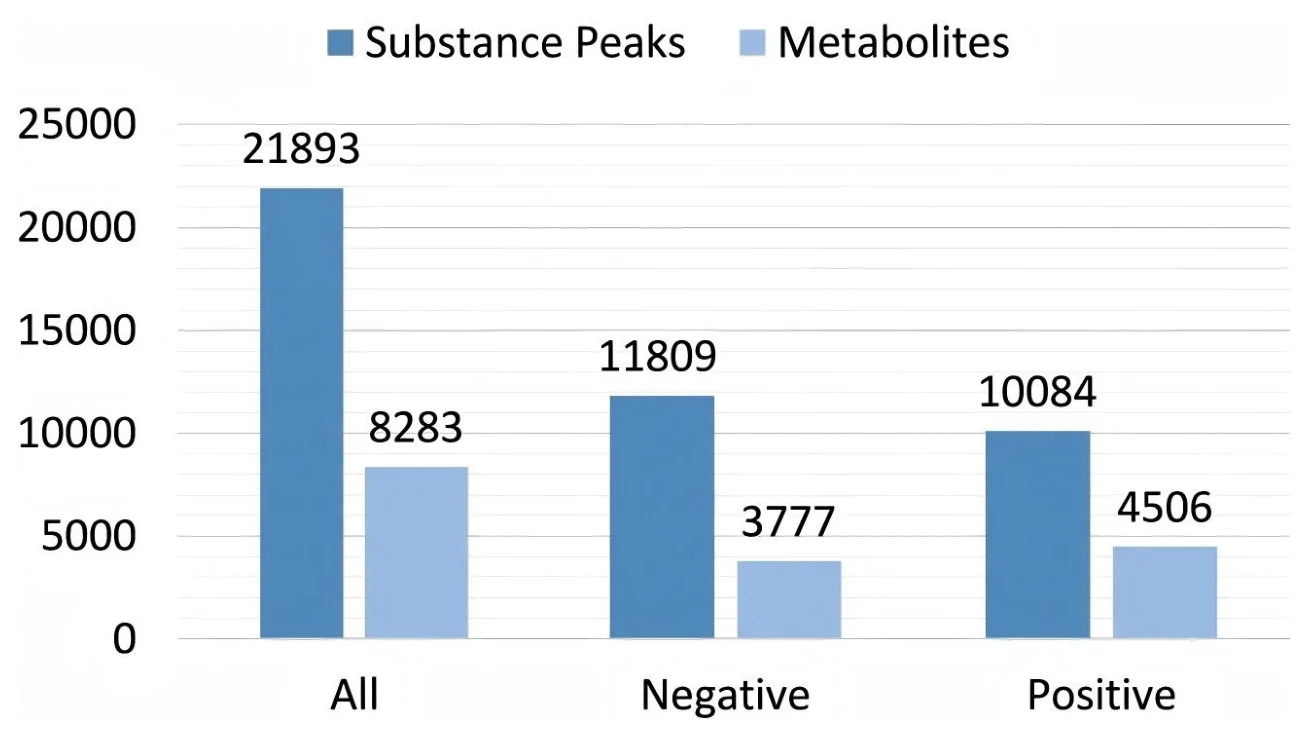
Statistical Chart of Substance Peaks and Metabolites.

The complementary information obtained from positive- and negative-ion acquisition increased the overall metabolome coverage. A total of 3777 putatively annotated metabolites were obtained from 11,809 detected features in negative-ion mode, whereas 4506 putatively annotated metabolites were obtained from 10,084 detected features in positive-ion mode (Figure 1). These results indicate that the combined acquisition modes provided broad coverage of the soybean metabolome.

**Figure 2:**
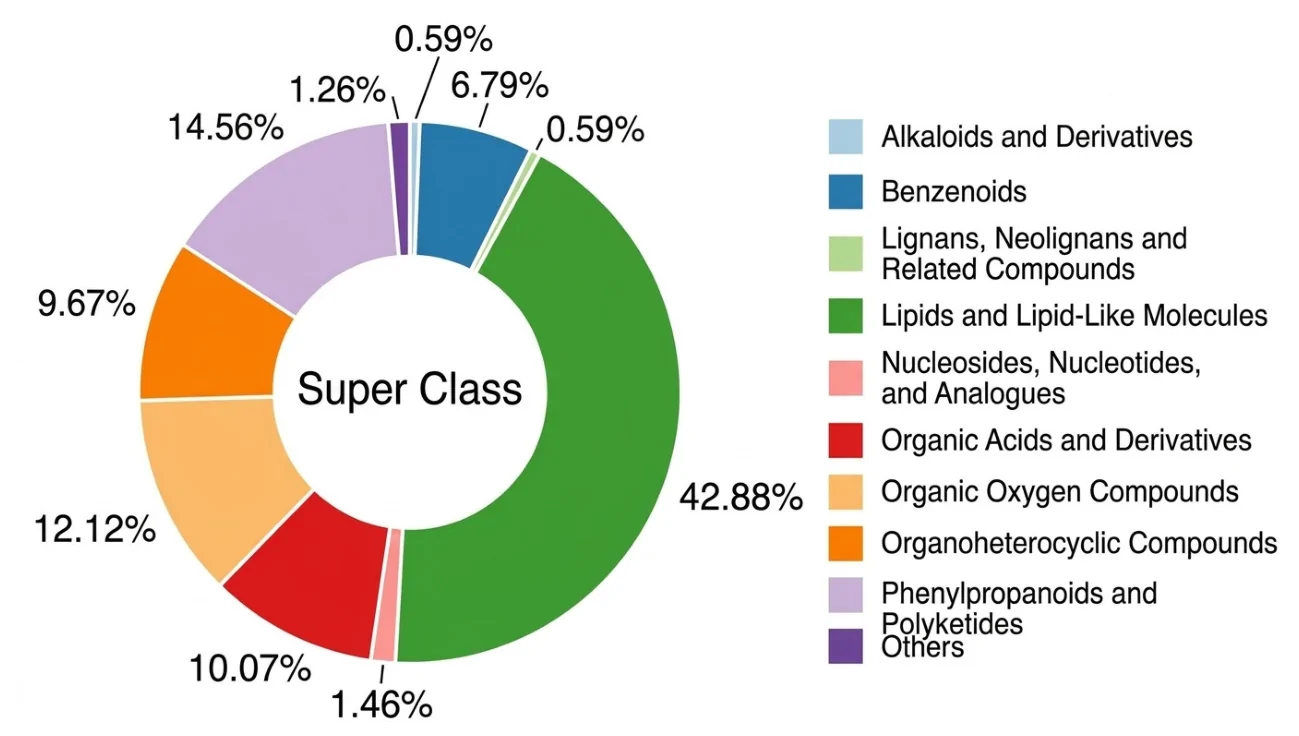
Composition Chart of Metabolite Superclasses.

The putatively annotated metabolites exhibited significant structural diversity. Excluding unclassified compounds, lipids and lipid-like molecules constituted the largest superclass (42.88%), serving as fundamental components of biological membranes and energy storage systems. This was followed by phenylpropanoids and polyketides (14.56%), organic oxygen compounds (12.12%), organic acids and their derivatives (10.07%), and organoheterocyclic compounds (9.67%). Other minor chemical classes, including lignans, alkaloids, and nucleosides, each accounted for less than 1.5% of the total metabolome (Figure 2).

### 2.2. Non-Targeted Multivariate Statistical Analysis

**Figure 3:**
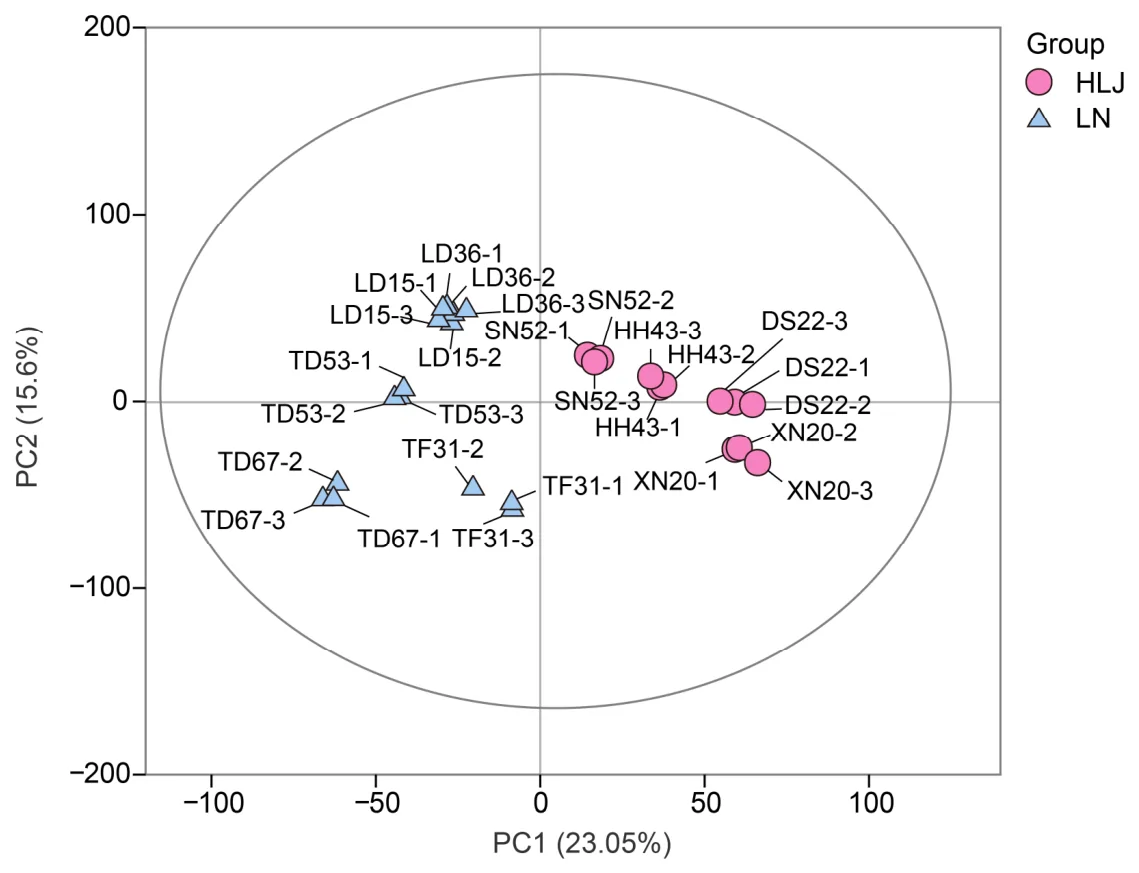
HLJ vs. LN PCA Score Plot.

Principal component analysis (PCA, unit-variance scaled, retaining two principal components) was first performed as an unsupervised approach to assess the overall structure of the metabolomic dataset and verify analytical reproducibility. The first two principal components, PC1 and PC2, explained 23.05% ($R^{2}X_{1}=0.2305$) and 15.60% ($R^{2}X_{2}=0.1560$) of the total variance, respectively, accounting for a cumulative variance explanation of 38.65% ($R^{2}X\left(\mathrm{cum}\right)=0.387$). The samples were generally distributed within the 95% confidence ellipse, with no obvious extreme outliers observed in the PCA score plot (Figure 3). Notably, the soybean samples from Heilongjiang (HLJ) and Liaoning (LN) exhibited a clear tendency toward separation along PC1. The HLJ samples were mainly distributed on the positive side of PC1, whereas the LN samples were predominantly located on the negative side. Furthermore, differences in within-group dispersion were observed, as HLJ cultivars clustered more tightly while LN cultivars displayed a broader distribution along PC2, suggesting greater intra-group metabolic variation in the LN cohort. In addition, following QC filtering (RSD≤30%), the three independent composite analytical replicates (−1, −2, and −3) within each of the nine cultivar–location groups clustered tightly together in the PCA score space, with a mean within-cultivar pairwise Euclidean distance of only 6.03±3.69 (median 5.50) compared to 76.75±32.22 (median 78.72) between cultivar centroids (a 12.7-fold ratio), confirming high full-workflow analytical reproducibility across sample extraction and LC-MS acquisition.

**Figure 4:**
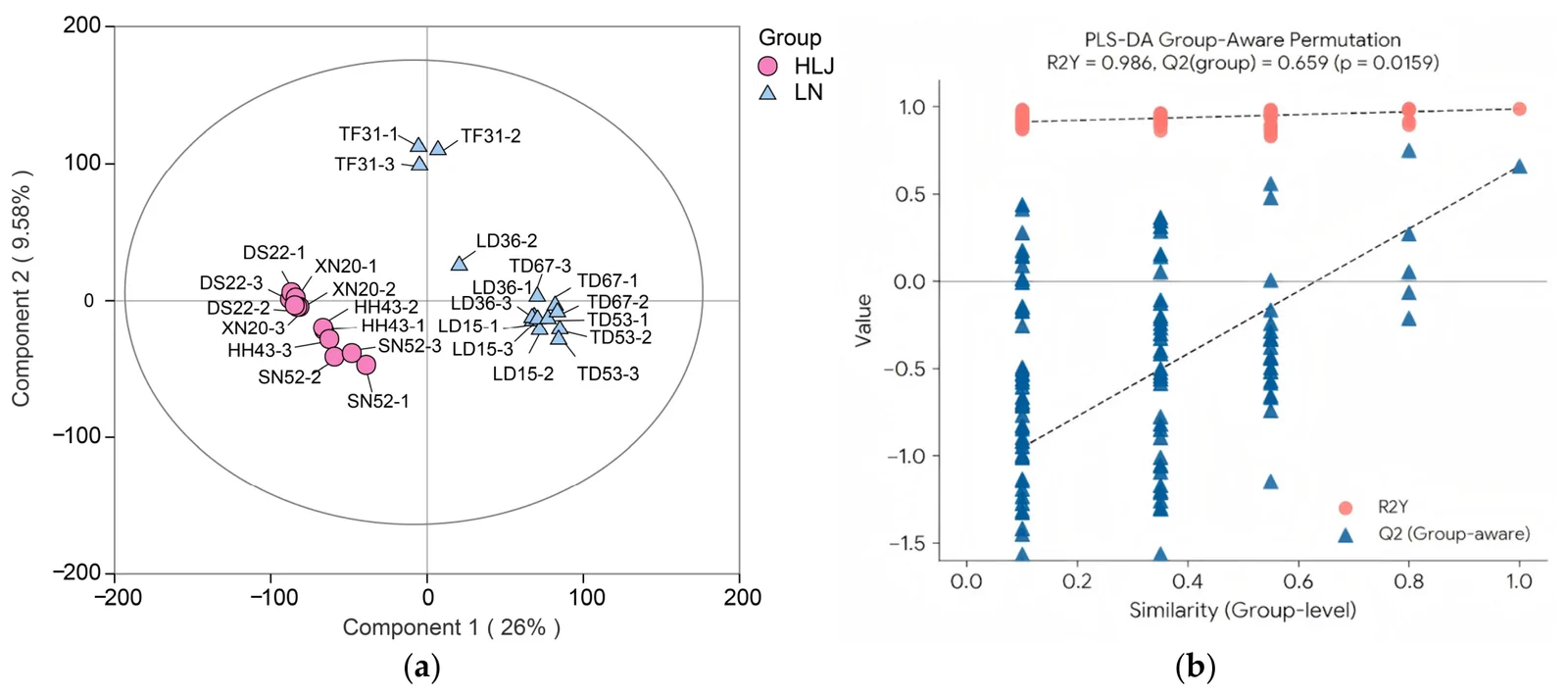
Partial least squares discriminant analysis (PLS-DA) of soybean samples from Heilongjiang and Liaoning provinces. (**a**) PLS-DA score plot demonstrating spatial separation between regional groups. (**b**) Group-restricted permutation test plot (126 exact group assignments) under 9-fold leave-cultivar-group-out cross-validation, assessing model robustness and the absence of overfitting at the true experimental unit level ($Q_{group}^{2}=0.659$, *p* = 0.0159).

To further evaluate the separation between the two regions, partial least squares discriminant analysis (PLS-DA, ${log}_{10}$-transformed and Pareto-scaled, retaining two latent components: p1 and p2) was subsequently performed (Figure 4a). Specifically, the first latent component (p1) explained 26.0% of the predictor variance ($R^{2}X_{p1}=0.260$, with $R^{2}Y_{p1}=0.851$ and $Q_{p1}^{2}=0.795$), and the second component (p2) explained an additional 9.58% ($R^{2}X_{p2}=0.0958$, with $R^{2}Y_{p2}=0.135$ and component $Q_{p2}^{2}=0.751$), resulting in a cumulative $R^{2}X\left(\mathrm{cum}\right)$ of 0.356 (35.58%) (Figure S3a). Compared with the unsupervised PCA model, the PLS-DA score plot achieved a more pronounced separation, with HLJ samples distributed in the negative quadrant and LN samples located in the positive quadrant along Component 1. However, because subsamples drawn from the same seed lot are naturally clustered, standard subsample-level cross-validation overestimates predictive performance. Therefore, a strict group-aware strategy (9-fold leave-cultivar-group-out cross-validation) was adopted as the primary validation approach. Under this rigorous validation, the PLS-DA model yielded a conservative group-level predictive accuracy of $Q_{group}^{2}=0.659$, with exact group-restricted permutation testing confirming model robustness (*p* = 0.0159, Figure 4b).

**Figure 5:**
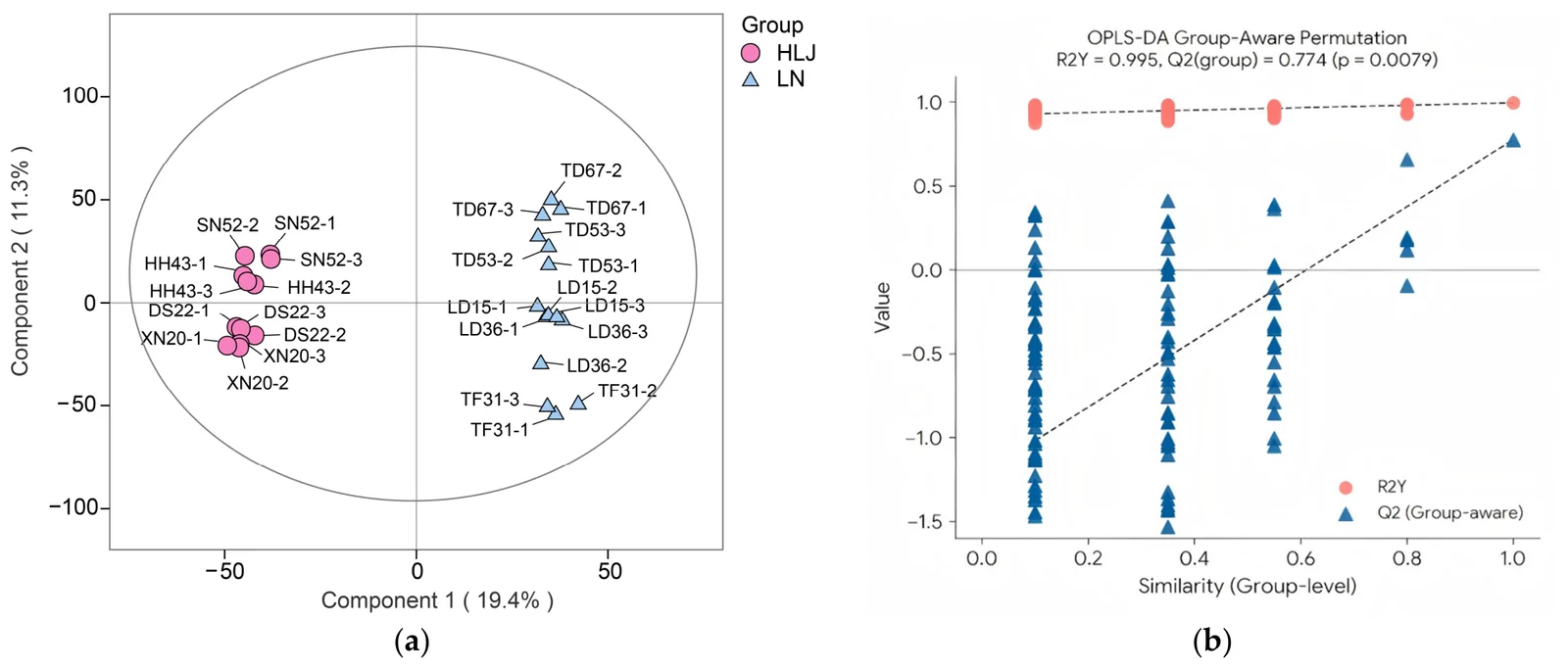
Orthogonal partial least squares discriminant analysis (OPLS-DA) of soybean samples from Heilongjiang and Liaoning provinces. (**a**) OPLS-DA score plot illustrating complete spatial separation along the predictive component. (**b**) Group-restricted permutation test plot (126 exact group assignments) under 9-fold leave-cultivar-group-out cross-validation, confirming high predictive accuracy at the true experimental unit level ($Q_{group}^{2}=0.774$, *p* = 0.0079).

OPLS-DA (${log}_{10}$-transformed and unit-variance scaled, retaining one predictive component p1 and one orthogonal component o1) was further applied to distinguish predictive variation associated with the regional grouping from orthogonal variation (Figure 5a). The predictive component (p1) explained 19.4% of the total predictor variance ($R^{2}X_{p1}=0.194$, capturing $R^{2}Y_{p1}=0.941$ and $Q_{p1}^{2}=0.896$), while the first orthogonal component (o1) accounted for 11.3% of the variance ($R^{2}X_{o1}=0.113$, with $R^{2}Y_{o1}=0.0541$ and $Q_{o1}^{2}=0.0663$), yielding a cumulative $R^{2}X\left(\mathrm{cum}\right)$ of 0.307 (30.7%) (Figure S3b). The OPLS-DA score plot showed a clear separation between the HLJ and LN samples along the predictive component, with HLJ samples distributed on the left side of the origin and LN samples on the right. Employing the same strict group-aware validation strategy, the OPLS-DA model yielded a robust group-level predictive accuracy of $Q_{group}^{2}=0.774$ (out-of-group accuracy = 96.3%, 26/27 samples). The exact group-restricted permutation test confirmed the validity and high predictive accuracy of the model (*p* = 0.0079, Figure 5b).

Notably, across all three score plots (Figure 3, Figure 4a and Figure 5a), the composite subsamples (−1, −2, and −3) drawn from the same harvested seed lot clustered tightly together, indicating high within-lot homogeneity. Although the strict group-aware cross-validation yielded more conservative predictive statistics compared to subsample-level estimations, both $Q_{group}^{2}$ values remained well above the 0.50 threshold. Prediction residuals were mainly attributable to the borderline LN cultivar TF31. Together, the unsupervised PCA and rigorously validated PLS-DA/OPLS-DA results consistently demonstrated pronounced differences in the metabolomic profiles across the nine sampled cultivar–location experimental units.

### 2.3. Screening and Clustering Analysis of Differential Metabolites

Univariate statistical analysis, incorporating Fold Change (FC) calculations and Student’s *t*-tests, was initially performed across the subsample replicates to evaluate the variation in each metabolite between the Heilongjiang and Liaoning groups. The *p*-value was utilized to determine the statistical significance of the abundance differences, while the FC value—defined explicitly as the ratio of the arithmetic mean normalized peak intensity of the HLJ group to that of the LN reference group ($\mathrm{FC}={\mathrm{Mean}}_{\mathrm{HLJ}}/{\mathrm{Mean}}_{\mathrm{LN}}$, with $\log_{2}\mathrm{FC}=\log_{2}\left[{\mathrm{Mean}}_{\mathrm{HLJ}}/{\mathrm{Mean}}_{\mathrm{LN}}\right]$)—quantified the magnitude and direction of these changes. Although univariate analysis provides direct comparative metrics, relying on it alone for high-dimensional metabolomics data lacks global perspective and is susceptible to false positives [23]. Therefore, this study additionally applied the Benjamini–Hochberg False Discovery Rate (FDR) correction to adjust the statistical significance. To extract the core differential metabolites driving the regional divergence, a multi-dimensional screening strategy integrating univariate statistics with the multivariate OPLS-DA model was applied [24]. Metabolites were designated as exploratory candidate differential features if they satisfied three thresholds: a Variable Importance in Projection (VIP) score > 1.0, subsample-level FDR < 0.05, and an FC > 2.0 ($\log_{2}\mathrm{FC}>1.0$) or < 0.5 ($\log_{2}\mathrm{FC}<-1.0$).

**Figure 6:**
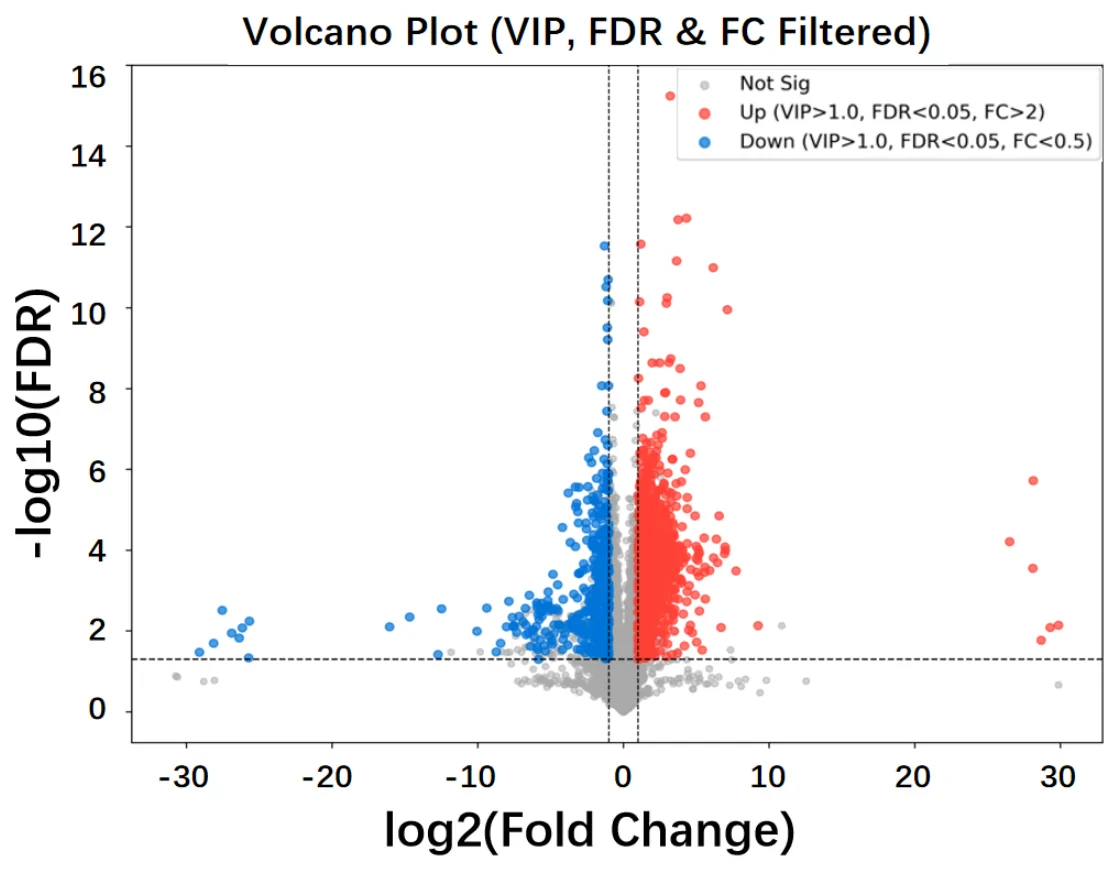
Volcano plot of exploratory candidate differential metabolites between Heilongjiang (HLJ) and Liaoning (LN) soybean groups based on multi-dimensional screening ($\mathrm{FC}={\mathrm{Mean}}_{\mathrm{HLJ}}/{\mathrm{Mean}}_{\mathrm{LN}}$). Red dots (labeled “Up”) represent metabolites with significantly higher abundance in HLJ relative to LN (n=953; VIP>1.0, FDR<0.05, and FC>2.0), blue dots (labeled “Down”) indicate metabolites with significantly lower abundance in HLJ relative to LN (n=532; VIP>1.0, FDR<0.05, and FC<0.5), and gray dots signify non-significant features. Note that the extreme points at $|\log_{2}\mathrm{FC}|>25$ (n=14) reflect region-specific (“all-or-none”) features completely undetected in one group and imputed with the global half-minimum baseline ($2.50\times 10^{-5}$); sensitivity values under feature-wise half-minimum imputation are detailed in Supplementary Table S1.

Volcano plot analysis (Figure 6) visually captures this initial screening process. In the global comparison between the HLJ and LN groups, a total of 1485 putatively annotated metabolites (MSI Level 2/3) met the screening criteria, comprising 953 metabolites with significantly higher abundance ($\mathrm{FC}>2.0,\log_{2}\mathrm{FC}>1.0$; red nodes) and 532 metabolites with significantly lower abundance ($\mathrm{FC}<0.5,\log_{2}\mathrm{FC}<-1.0$; blue nodes, corresponding to higher abundance in LN) in HLJ soybeans relative to LN soybeans (Supplementary Table S1). This asymmetrical quantitative distribution (953 vs. 532) indicates that the sampled HLJ soybean varieties exhibit higher metabolite abundances across a broader range of primary and secondary metabolic features. Regarding missing-value distribution and its impact on fold-change magnitudes, across the 1485 differential metabolites (40,095 data cells), the overall missingness rate was 9.54% (median = 0.00%), with 1016 metabolites (68.42%) detected across all 27 samples (0% missing values). Evaluation of the volcano plot (Figure 6) revealed that the extreme fold-change outliers ($|\log_{2}\mathrm{FC}|>10$, n=19, including 14 features with $|\log_{2}\mathrm{FC}|\approx 25–30$) were driven by region-specific absence (6 features with 15/15 missing values in LN and 8 features with 12/12 missing values in HLJ) where the absent group was imputed with the global half-minimum baseline ($2.50\times 10^{-5}$). Sensitivity re-evaluation using feature-wise (per-metabolite) half-minimum imputation compressed these extreme ratios to moderate magnitudes while confirming that 1479 of the 1485 metabolites (99.60%; 951 higher abundance and 528 lower abundance in HLJ) still strictly satisfied FC>2.0 or <0.5 (Supplementary Table S1). Furthermore, to distinguish higher-confidence structural matches from ambiguous database assignments in Supplementary Table S1, each differential feature was stratified by its MS/MS fragmentation support into MSI Level 2 putative annotations (supported by experimental MS/MS fragmentation spectra, Fragmentation Score > 0) and MSI Level 3 tentative annotations (matched solely by MS1 accurate mass and isotopic formula without MS/MS fragmentation, Fragmentation Score = 0, where non-plant database names reflect isobaric formula isomers). Accordingly, only biologically plausible endogenous phytochemicals—specifically MSI Level 2 annotations with robust MS/MS fragmentation scores—were prioritized for downstream biological interpretation. However, because the replicates within each variety are subsamples drawn from the same harvested seed lot provided by the local seed station, samples are clustered within cultivar and location. Treating all subsample observations as fully independent in univariate tests leads to pseudoreplication and underestimates uncertainty (inflating statistical significance). When we accounted for this dependence by aggregating the nested subsamples to their respective cultivar–location means (n=4 HLJ vs. n=5 LN independent experimental units), statistical significance was naturally more conservative due to the smaller number of true experimental units (N=9); nevertheless, the primary discriminative metabolite classes—most notably isoflavonoids, triterpenoid saponins, unsaturated fatty acyls, sphingolipids, and tryptophan derivatives—consistently maintained large fold-change magnitudes (FC>2.0 or <0.5) and clear divergence across the cultivar–location means. However, because the true independent experimental units are limited to the nine cultivar–location means (N = 9), rigorous metabolite-wise univariate inference with multiple-testing correction lacks statistical power at the group level. Therefore, these 1485 features are strictly defined as exploratory candidate differential metabolites rather than statistically significant differential metabolites.

**Figure 7:**
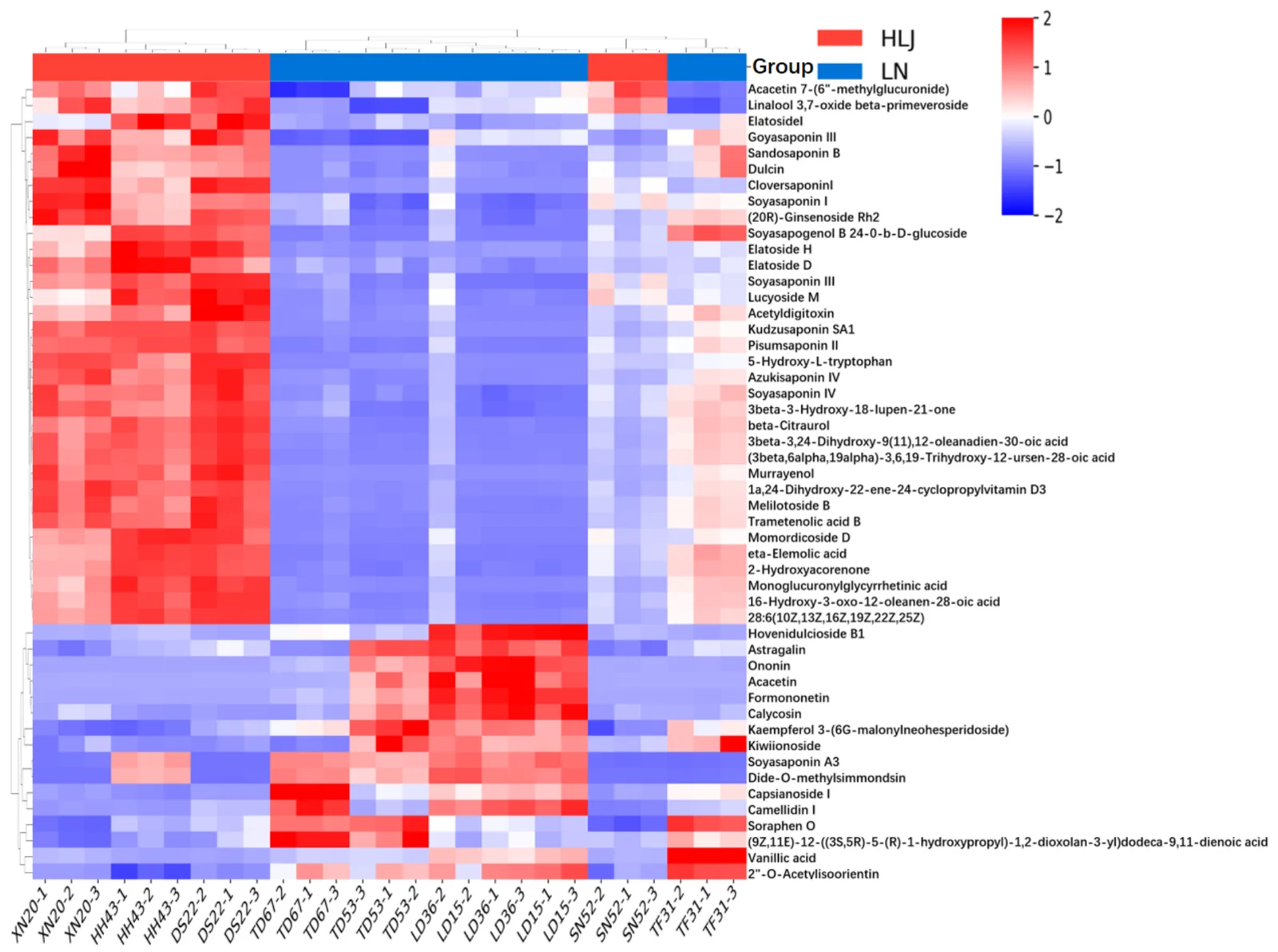
Hierarchical clustering heatmap of the top 50 differential metabolites ranked by VIP scores across HLJ and LN soybean samples. The color scale indicates relative metabolite abundance, with red and blue representing high and low accumulation levels, respectively. Note that because these metabolites were pre-selected for regional discrimination, the sample clustering serves as a visualization of the selected discriminatory variables rather than an independent confirmation of group separation.

Hierarchical clustering heatmap analysis was subsequently performed on the top 50 differential metabolites, ranked by VIP scores, to visualize their relative abundance patterns across all samples (Figure 7). Crucially, because these 50 metabolites were pre-selected using supervised OPLS-DA (VIP>1.0) and univariate thresholds (FDR<0.05, FC>2.0 or <0.5) specifically to discriminate between the HLJ and LN groups, the resulting sample dendrogram primarily provides a visualization of the selected discriminatory variables rather than an independent confirmation of regional separation (whereas unbiased global structure is assessed by unsupervised PCA in Figure 3, and model generalizability is evaluated via cultivar-grouped cross-validation in Section 2.2). Within this visualization of the selected discriminatory features, the dendrogram at the top shows a macro-level bifurcation in which the majority of Heilongjiang (HLJ) varieties (XN20, HH43, DS22) and Liaoning (LN) varieties group into two main clades with contrasting metabolite abundance patterns. However, even across these top-ranked regionally discriminatory variables, the regional grouping is not strictly absolute. For instance, the HLJ variety SN52 clusters within the broader LN branch based on its metabolic similarity, and the LN variety TF31 also exhibits localized divergence from other LN cultivars. This moderate inter-group overlap illustrates that sample clustering in the heatmap is not solely defined by regional cohort, suggesting that cultivar-specific genetic differences also contribute substantially to local metabolic heterogeneity.

Vertically, the metabolites are distinctively divided into two inverse accumulation modules. Crucially, the key biomarkers highlighted in our biological discussion below are MSI Level 2 putative annotations supported by high-quality experimental MS/MS fragmentation spectra (Supplementary Table S1). The upper module is highly abundant (dark red) in typical HLJ samples, predominantly enriched in triterpenoid saponins and their derivatives, including Soyasaponin I (overall score = 57.3, MS/MS fragmentation score = 89.8), Soyasaponin III (score = 57.4, MS/MS = 90.3), Soyasaponin IV (score = 57.1, MS/MS = 94.8), Sandosaponin B (score = 56.6, MS/MS = 85.8), Cloversaponin I (score = 57.3, MS/MS = 87.7), and Elatoside I (score = 54.9, MS/MS = 89.6). Conversely, the lower module is enriched in LN samples, primarily comprising flavonoids and isoflavones such as Formononetin (score = 57.7, MS/MS = 91.2), Ononin (score = 55.5, MS/MS = 86.0), Astragalin (score = 57.3, MS/MS = 94.5), Calycosin (score = 57.2, MS/MS = 89.3), and Acacetin (score = 58.5, MS/MS = 95.3). Concurrently, the outlier cultivars exhibit highly specific localized metabolic fluctuations. For instance, Vanillic acid (score = 56.6, MS/MS = 91.2) exhibits a distinctly high relative abundance in the divergent LN cultivar TF31; meanwhile, Kaempferol 3-(6G-malonylneohesperidoside) (score = 57.5, MS/MS = 92.1) displays significantly lower accumulation levels in the crossover HLJ cultivar SN52 compared to other groups. This contrasting overall metabolic profile, combined with the specific accumulation patterns in individual cultivars, indicates that the observed metabolomic variation reflects the combined influences of regional growing conditions and cultivar-specific genetic backgrounds across the sampled cultivar–location combinations.

Existing literature confirms that metabolic profiling is a powerful approach for investigating geographical variations in agricultural products. Previous studies have shown that metabolic differences in crops are shaped by the combined effects of environmental factors—such as soil nutrients, temperature regimes, and solar radiation across different latitudes—and cultivar genetic backgrounds [25,26,27]. For example, secondary metabolites such as flavonoids and isoflavones are known to be responsive to both varietal genetics and abiotic factors like temperature and light intensity, making them informative markers for characterizing regional germplasm [28]. Similarly, studies on leguminous and cereal crops have demonstrated that lipid composition and triterpenoid saponin accumulation vary substantially across different ecological zones and genotypes [29]. Furthermore, recent multi-omics investigations have successfully utilized amino acid derivatives and phenolic compounds to differentiate plant materials from distinct geographical origins [30,31,32]. Building upon this literature, our screening of 1485 putatively annotated differential metabolites, coupled with the unsupervised PCA structure and cultivar-grouped cross-validated multivariate models (with the abundance patterns of the top selected discriminatory features visually summarized in Figure 7), demonstrates pronounced regional and cultivar-associated variation in soybean metabolite accumulation patterns between the sampled HLJ and LN cohorts. However, any claim regarding geographic authentication requires strict qualification: because the present dataset is limited to nine cultivars from single sites, single seed lots, and a single harvest year, the differential metabolites identified here (such as specific soyasaponins and isoflavonoids) should be regarded strictly as preliminary candidate markers rather than validated geographic biomarkers. Independent validation across multiple harvest years, diverse sampling sites within each province, multiple independent seed lots, and expanded cultivar panels will be essential to confirm their stability and generalizability before practical geographic authentication can be claimed.

### 2.4. Exploratory KEGG Pathway Analysis

**Figure 8:**
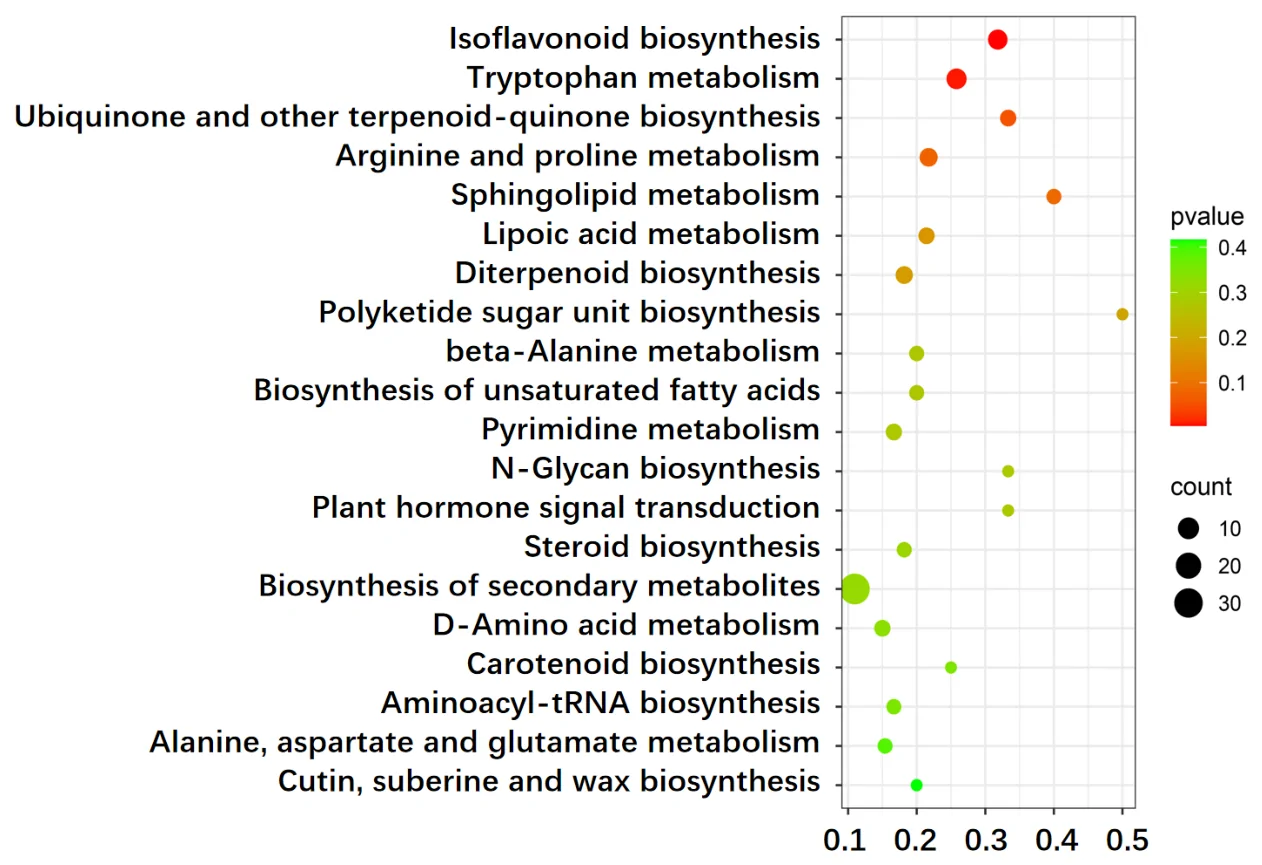
Exploratory KEGG pathway enrichment analysis of differentially accumulated metabolites between HLJ and LN soybean groups (top 20 pathways ranked by nominal p-value). The bubble size indicates the number of differential metabolites mapped to each pathway, and the color scale represents the unadjusted (nominal) p-value (note: no pathway reached an FDR-adjusted q<0.05; see Supplementary Table S2).

To explore the broader biochemical context of the putatively annotated differential metabolites, we mapped them to the KEGG database for hypergeometric pathway over-representation analysis. The detailed statistical results across all 61 mapped pathways (81 annotated differential metabolites mapped against 794 background KEGG compounds) are provided in Supplementary Table S2. Crucially, a clear distinction must be made between unadjusted nominal p-values and results corrected for multiple testing (FDR-adjusted q-values). Because only a subset of untargeted LC-MS features possess formal KEGG compound identifiers, statistical power across the 61 pathways was constrained, and none of the pathways remained statistically significant after multiple-testing correction at an FDR threshold of q<0.05 (minimum q=0.261; Supplementary Table S2). Therefore, the KEGG pathway analysis is presented here as an exploratory and hypothesis-generating framework—rather than a confirmatory test—to organize the individually significant differential metabolites into functional biochemical modules. Based on nominal p-values, only two pathways achieved nominal significance (p<0.05): isoflavonoid biosynthesis (ko00943, 7 mapped metabolites, nominal p=0.0043, q=0.261) and tryptophan metabolism (ko00380, 8 mapped metabolites, nominal p=0.0093, q=0.283), while three additional pathways showed marginal nominal trends (0.05≤p<0.10), including ubiquinone and other terpenoid-quinone biosynthesis (ko00130, p=0.0547, q=0.928), arginine and proline metabolism (ko00330, p=0.0754, q=0.928), and sphingolipid metabolism (ko00600, p=0.0839, q=0.928) (Figure 8). In the isoflavonoid biosynthesis pathway, the sampled LN soybean cultivars exhibited significantly higher steady-state abundances of isoflavones and their derivatives compared to the HLJ group. Additionally, from a descriptive pathway-mapping perspective, the overarching biosynthesis of secondary metabolites pathway (ko01110) captured the largest absolute count of differential features (35 mapped metabolites, nominal p=0.318, q=0.928), reflecting substantial variation in secondary metabolite pool sizes between the sampled HLJ and LN cultivar–location groups. In lipid-related pathways, including sphingolipid metabolism and the biosynthesis of unsaturated fatty acids (ko01040, nominal p=0.272, q=0.928), the individual differential metabolites mapped to these routes showed higher steady-state abundances of lipid molecules and fatty acyls in HLJ soybeans; because neither membrane biophysical properties nor site-specific temperature records were directly measured in this study, any potential connection between these lipid differences and cellular membrane fluidity under high-latitude cold conditions is proposed strictly as an exploratory hypothesis. Furthermore, across tryptophan metabolism, arginine and proline metabolism, and ubiquinone and other terpenoid-quinone biosynthesis, the two geographic groups exhibited differential steady-state accumulation of specific amino acid and quinone derivatives, highlighting distinct regional and cultivar-associated metabolic profiles across the sampled soybean cohorts.

**Figure 9:**
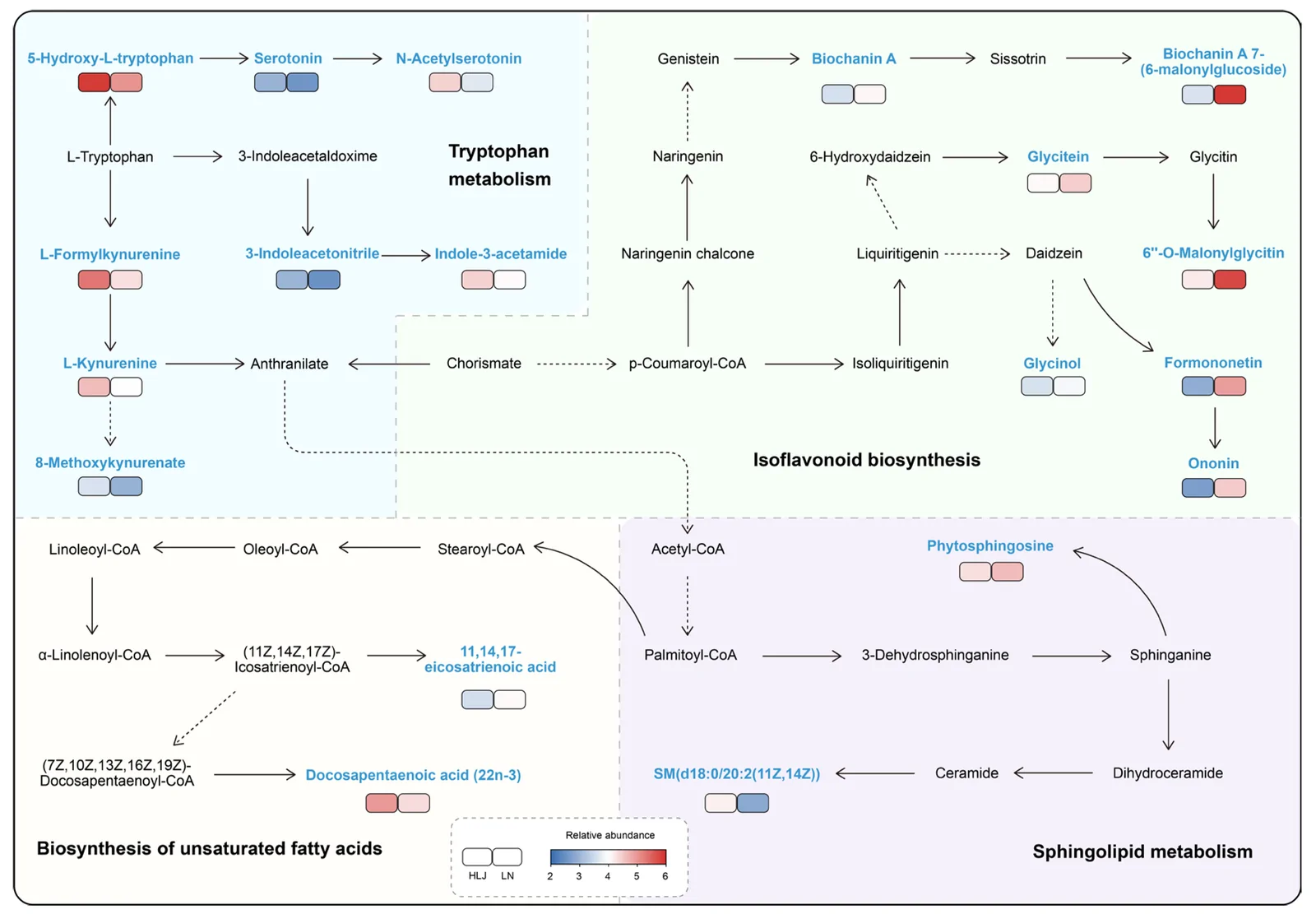
Comprehensive metabolic network mapping of key differentially accumulated metabolites between Heilongjiang (HLJ) and Liaoning (LN) soybean cultivars. The reconstructed pathways delineate specific steady-state abundance variations across tryptophan metabolism, isoflavonoid biosynthesis, biosynthesis of unsaturated fatty acids, and sphingolipid metabolism. Solid arrows indicate direct enzymatic steps in reference pathways, while dashed arrows represent multi-step conversions (note: arrows represent canonical pathway topology rather than measured metabolic flux). The dual-box heatmap beneath specific metabolites indicates their relative steady-state abundance in the HLJ group (left box) and the LN group (right box), with a color scale from blue (low abundance) to red (high abundance). Metabolites supported by experimental MS/MS fragmentation (MSI Level 2) include Formononetin (MS/MS=91.2), Ononin (86.0), 5-Hydroxy-L-tryptophan (81.8), Docosapentaenoic acid (75.2), Biochanin A 7-(6-malonylglucoside) (72.7), Indole-3-acetamide (72.7), 6″-O-Malonylglycitin (63.0), Phytosphingosine (60.9), L-Formylkynurenine (58.9), Glycitein (54.8), 11,14,17-Eicosatrienoic acid (51.7), and N-Acetylserotonin (12.8), whereas nodes matched solely by MS1 accurate mass and isotope distribution (Fragmentation Score = 0: Serotonin, 3-Indoleacetonitrile, 8-Methoxykynurenate, Biochanin A, Glycinol, and SM(d18:0/20:2)) represent MSI Level 3 tentative annotations.

To intuitively visualize the pathway-level differences and specific steady-state abundance changes within these key candidate pathways, a schematic metabolic network map of representative differential metabolites (each individually satisfying VIP>1.0, FDR<0.05, and FC>2.0 or <0.5) was constructed (Figure 9). This mapping visually summarizes the distinct steady-state metabolic signatures of the two regional groups, reflecting variations associated with the sampled cultivar–location combinations. As illustrated in the network, the Liaoning group exhibits higher steady-state abundances of downstream isoflavones and their diverse malonylated and glycosylated derivatives. Conversely, the Heilongjiang group exhibits substantially higher steady-state abundances of specific structural lipids, including unsaturated fatty acyls and complex sphingolipids, alongside higher levels of tryptophan-derived indoleamines. Although these static pool-size differences do not directly measure dynamic pathway flux, they illustrate pronounced divergence in steady-state metabolite profiles between the sampled HLJ and LN soybean cohorts, which may be linked to both local environmental differences and the genetic characteristics of the respective cultivars.

A prominent distinguishing feature of the LN soybean group at the individual metabolite level is the higher steady-state abundance of isoflavones, flavonoids, and complex secondary glycosides, including Formononetin (MS/MS=91.2), Ononin (MS/MS=86.0), Calycosin (MS/MS=89.3), Biochanin A 7-(6-malonylglucoside) (MS/MS=72.7), 6″-O-Malonylglycitin (MS/MS=63.0), Glycitein (MS/MS=54.8), Orobol 7-O-(6″-malonylglucoside) (MS/MS=53.1), and Capsianoside I (MS/MS=85.1) (each individually satisfying VIP>1.0, FDR<0.05, and FC<0.5; Figure 7 and Figure 9). Consistent with these individual metabolite differences, our exploratory KEGG analysis indicated nominal enrichment in the “Isoflavonoid biosynthesis” pathway (p=0.0043, q=0.261) and extensive feature mapping within “Biosynthesis of secondary metabolites” (p=0.318, q=0.928). Extensive research has demonstrated that isoflavones are important secondary metabolites that function as phytoestrogens and antioxidants [33,34]. Beyond their roles in plant defense, existing literature highlights their potential health-promoting effects in humans, including anti-inflammatory, cardioprotective, and cholesterol-lowering activities [35,36]. From an ecophysiological perspective, previous studies indicate that ultraviolet radiation and thermal regimes can act as environmental signals to induce chalcone synthase (CHS) and isoflavone synthase (IFS) transcription, thereby enhancing antioxidant capacity under warm conditions [37,38,39]. At a macroclimatic level, Liaoning province is located at a lower latitude and generally experiences warmer growing-season temperatures than Heilongjiang; however, because site-specific meteorological parameters (such as daily temperature and UV radiation) were not directly measured at the sampling plots, environmental correlations cannot be directly tested in our dataset. Therefore, while the consistently higher steady-state abundance of multiple isoflavonoids in the LN cohort aligns with published literature models, we propose strictly as a tentative working hypothesis that these elevated isoflavone pools may be partly associated with the warmer regional climate alongside the inherent genetic background of the locally bred LN cultivars.

Under low-temperature stress, plant cell membranes are susceptible to increased rigidity, which can affect ion transport and membrane-associated proteins [40]. To mitigate these effects, previous physiological studies have shown that cold-tolerant plants can adjust the unsaturation of fatty acids and modulate sphingolipid composition to maintain membrane stability and cold acclimation signaling [41,42]. In our dataset, although lipid-related KEGG pathways—such as sphingolipid metabolism (p=0.0839, q=0.928) and biosynthesis of unsaturated fatty acids (p=0.272, q=0.928)—did not reach pathway-level significance after FDR correction, inspection of individual differential features (FDR<0.05) revealed that soybeans sampled from HLJ exhibit higher steady-state abundances of specific unsaturated fatty acyls (e.g., docosapentaenoic acid [22n-3], Score=53.2, MS/MS=75.2; and 11,14,17-eicosatrienoic acid, Score=48.9, MS/MS=51.7), sphingolipid metabolites (e.g., phytosphingosine, Score=50.1, MS/MS=60.9, alongside the MSI Level 3 tentative MS1 match SM(d18:0/20:2), Score=37.0), and glycerophospholipids (Figure 9). Furthermore, existing literature indicates that black soil agroecosystems in Northeast China generally provide fertile nutrient conditions for primary and secondary metabolism [43,44,45]. Nevertheless, because direct measurements of field temperature, soil nutrient composition, and membrane biophysical properties were not available in this study, these macro-environmental descriptions serve only as general geographical context [46]. Accordingly, the elevated steady-state abundances of these specific lipid species in the HLJ cohort are presented cautiously as a hypothesis-generating observation—potentially reflecting cold-tolerant cultivar genetics and/or regional environmental adaptation—that requires future verification using direct environmental monitoring and physiological assays.

Beyond secondary phenolics and lipid species, our exploratory pathway analysis showed nominal enrichment in tryptophan metabolism (p=0.0093, q=0.283), driven by individually significant differential metabolites (FDR<0.05). Previous studies have shown that tryptophan serves as a precursor for indoleamines such as serotonin and melatonin [47]. In this study, specific tryptophan derivatives supported by MS/MS fragmentation—most notably 5-hydroxy-L-tryptophan (Score=55.4, MS/MS=81.8), indole-3-acetamide (Score=53.5, MS/MS=72.7), L-formylkynurenine (Score=50.7, MS/MS=58.9), and N-acetylserotonin (Score=40.4, MS/MS=12.8, alongside the tentative MS1 match serotonin, Score=37.6)—exhibited higher steady-state abundance in the Heilongjiang group. In the literature, N-acetylserotonin is known as the biochemical precursor to melatonin, an endogenous free radical scavenger implicated in mitigating chilling-induced reactive oxygen species (ROS) accumulation [48,49,50,51]. Because direct field chilling records, metabolic flux rates, and cellular ROS levels were not directly measured, we propose strictly as an exploratory hypothesis that the larger steady-state pools of indoleamine precursors observed in the sampled northern soybean cultivars may be associated with cold-tolerant germplasm traits or high-latitude growing conditions, providing a candidate biochemical target for future controlled-environment validation.

### 2.5. Limitations and Future Work

Several limitations of this study should be noted to guide future research. First, different region-specific cultivars were collected from a single site per province without direct meteorological measurements (e.g., daily temperature, solar radiation, precipitation) or soil physicochemical analyses during the growing season. Consequently, cultivar genotype and regional environment are statistically confounded, and references to regional climatic conditions remain qualitative macroclimatic descriptions. Disentangling the precise variance contributions of genotype (G), environment (E), and genotype × environment (G×E) interactions—as well as statistically testing direct climate–metabolite correlations—will require reciprocal transplant (common-garden) experiments or multi-year, multi-location factorial trials equipped with continuous environmental and soil monitoring. Second, the three replicates analyzed per cultivar were composite subsamples drawn from the same harvested seed lot provided by the respective seed station rather than independent field-plot biological replicates, and the sampling was conducted within a single harvest year. Consequently, the differential metabolites identified in this study represent preliminary candidate markers specific to the sampled cultivar–location cohorts rather than universally validated geographic biomarkers. Establishing validated biomarkers for practical geographic authentication will require rigorous independent validation using external cohorts spanning multiple harvest years, multiple geographic sites per province, independent commercial seed lots, and a broader panel of cultivars. Third, all metabolites reported in this study represent MSI Level 2/3 putative annotations based on spectral database matching rather than MSI Level 1 identifications confirmed with authentic standards, and none of the KEGG pathways survived multiple-testing correction at an FDR threshold of q<0.05 (Supplementary Table S2), rendering the pathway-level analyses exploratory rather than confirmatory. Furthermore, a fundamental distinction must be made between static metabolite abundance and dynamic metabolic flux: because untargeted metabolomics quantifies steady-state metabolite pool sizes at harvest rather than pathway turnover rates, and because direct environmental and physiological parameters were not measured, these data alone cannot directly demonstrate metabolic flux, carbon resource allocation, biophysical membrane-fluidity mechanisms, or direct causal climate-adaptation mechanisms. Accordingly, all proposed links between regional climatic conditions and specific metabolic pathways—including lipid-associated membrane adaptation, isoflavone-related thermal protection, and indoleamine-mediated antioxidant defense—are presented strictly as tentative, literature-informed working hypotheses. Future studies incorporating authentic reference standards (for MSI Level 1 confirmation), ^13^C/^15^N stable-isotope tracing (metabolic flux analysis), controlled-environment stress treatments with quantitative meteorological logging, transcriptomic and enzymatic assays, and direct biophysical/physiological measurements (such as membrane fluidity fluorescence polarization and cellular reactive oxygen species quantification) will be required to functionally verify these biochemical hypotheses.

## 3. Materials and Methods

### 3.1. Sample Collection

A total of 108 soybean seed subsamples (12 subsamples per variety) were obtained, comprising four varieties from Heilongjiang Province, China, and five from Liaoning Province, China. The specific varieties and sampling locations included: Xingnong 20 (XN20) from Beian City, Heilongjiang, China; Heihe 43 (HH43) from Nenjiang City, Heilongjiang, China; Dongsheng 22 (DS22) from Hailun City, Heilongjiang, China; Suinong 52 (SN52) from Bayan County, Heilongjiang, China; Liaodou 15 (LD15) from Xinmin City, Liaoning, China; Liaodou 36 (LD36) from Huludao City, Liaoning, China; Tiedou 53 (TD53) from Linghai City, Liaoning, China; Tiedou 67 (TD67) from Zhuanghe City, Liaoning, China; and Tiefeng 31 (TF31) from Pulandian District, Liaoning, China. Specifically, the harvested mature seed materials for each variety were provided by the respective seed stations affiliated with the local Academies of Agricultural Sciences in each production region. To clearly define the nature of the replication and the experimental unit, the 12 replicates per variety correspond to 12 subsamples randomly drawn from the same harvested seed material provided by the respective local seed station (yielding 108 subsamples in total). Prior to LC-MS extraction, every four subsamples within each variety were randomly pooled in equal weights to generate three composite analytical replicates per variety (designated as −1, −2, and −3 in the multivariate score plots and heatmaps) to reduce subsampling variability. Because the subsamples for each variety were drawn from the same harvested seed lot at a single location, the observations are clustered within cultivar and location; consequently, the nine cultivar–location seed lots (n=4 in HLJ and n=5 in LN) constitute the primary experimental units in this study. The geographical distribution of the sampling locations is illustrated in Figure S1, while detailed sample information, including variety codes, coordinates, and sample numbers, is summarized in Table S3.

### 3.2. Reagents, Instruments, and Equipment

Methanol, formic acid, acetonitrile, and deionized water were purchased from Thermo Fisher Scientific (Waltham, MA, USA). L-2-chlorophenylalanine, used as the internal standard, was obtained from Shanghai Hengchuang Biotechnology Co., Ltd. (Shanghai, China). All chemical reagents employed in this study were of analytical grade, while solvents used for liquid chromatography were of chromatographic grade to ensure analytical precision and consistency.

The experimental setup consisted of the following instruments: a fully automated rapid sample grinding system (Model Wonbio-E, Shanghai Wanbo Biotechnology Co., Ltd., Shanghai, China), an ultrasonic cleaning apparatus (Model F-060SD, Shenzhen Fuyang Technology Group, Shenzhen, China), a vortex mixer (Model TYXH-I, Shanghai Hanuo Instruments, Shanghai, China), a high-speed refrigerated centrifuge (Model TGL-16MS, Shanghai Luxiangyi Instruments, Shanghai, China), and an ultra-high-performance liquid chromatography (UHPLC) system (Dionex U3000, Thermo Fisher Scientific) coupled with a high-resolution mass spectrometer (Q Exactive series, Thermo Fisher Scientific).

Chromatographic separation was performed using a Waters ACQUITY UPLC HSS T3 column (100 mm × 2.1 mm, 1.8 μm particle size). This column provides excellent separation efficiency for complex biological matrices under UHPLC conditions and is particularly suitable for the retention and resolution of polar metabolites.

(Note: Because mature seeds were obtained from regional seed stations after harvest, direct site-specific meteorological records and soil physicochemical measurements during the growing season were not available; regional descriptions in this study refer to the general macroclimatic zones of the two collection areas.)

### 3.3. Sample Processing and Metabolite Extraction

#### 3.3.1. Sample Pretreatment

The 108 soybean seed subsamples were first cleaned rapidly with deionized water and 75% ethanol. Residual liquids were removed using sterile pipettes, and the samples were immediately immersed in liquid nitrogen for rapid freezing. Once completely frozen, the samples were transferred to a −80 °C freezer for long-term storage.

#### 3.3.2. Metabolite Extraction

For metabolite extraction, an 80 mg aliquot of each sample was mixed with 20 μL of internal standard (L-2-chlorophenylalanine, 0.06 mg/mL in methanol) and 1 mL of a methanol-water extraction solvent (7:3, *v*/*v*). After vortexing for 1 min, the mixture was homogenized using a 60 Hz grinder with steel balls at −20 °C for 2 min. The homogenate was sonicated in an ice-water bath for 30 min and incubated overnight at −20 °C. Following centrifugation (13,000 rpm, 10 min, 4 °C), 150 μL of the supernatant was filtered through a 0.22 μm organic-phase membrane and transferred to an LC injection vial for analysis. Quality Control (QC) samples were prepared by pooling equal aliquots from all sample extracts to monitor system stability.

#### 3.3.3. LC-MS Analysis

Metabolic profiling was performed on a Q Exactive™ HF-X mass spectrometer (Thermo Fisher Scientific, Waltham, MA, USA) coupled with a UHPLC system. Chromatographic separation was achieved using an ACQUITY UPLC HSS T3 column (100 mm × 2.1 mm, 1.8 μm) maintained at 40 °C. The mobile phases consisted of water containing 0.1% formic acid (mobile phase A) and acetonitrile containing 0.1% formic acid (mobile phase B). Mass spectrometry data were acquired under electrospray ionization (ESI) in both positive and negative ion scanning modes. To eliminate systematic run-order bias and avoid batch effects, all 27 composite study samples and quality control (QC) samples were analyzed within a single continuous analytical batch per ion mode in a completely randomized injection sequence generated across the HLJ and LN groups. Prior to sample acquisition, consecutive conditioning QC injections and procedural extraction blanks (prepared by processing the extraction solvent and internal standard through the identical grinding, sonication, centrifugation, and filtration workflow without soybean powder) were analyzed to equilibrate the system and establish baseline background signals, with pooled QC samples injected every 10 study samples throughout the sequence. The detailed LC gradient program and MS acquisition parameters are summarized in Table S4.

#### 3.3.4. Data Quality Assessment

Quality Control (QC) samples (QC1–QC5), prepared by pooling equal volumes of all sample extracts, were periodically analyzed (every 10 study samples) to monitor LC-MS system stability and assess within-batch signal drift. Because all samples were acquired within a single uninterrupted analytical batch per ion mode, inter-batch correction was not required; within-sequence signal drift and injection variations were corrected by normalizing peak intensities against the spiked internal standard (L-2-chlorophenylalanine) combined with Progenesis QI reference-run total abundance scaling. Across all analytical runs, the internal standard (L-2-chlorophenylalanine) demonstrated high chromatographic and mass spectrometric stability, exhibiting retention-time shifts |ΔRT|<0.10 min, mass accuracy within ±3 ppm, and raw peak-area variation (RSD) < 10%. Furthermore, to eliminate residual drift-sensitive features, ion peaks with an RSD>30% across the periodic pooled QC injections were strictly removed, ensuring that 100% of the 8283 retained features satisfied QC RSD≤30%. The ${log}_{10}$-transformed signal intensity distribution of the retained metabolites was first evaluated using box plots (Figure S2a). As shown in Figure S2a, the post-normalization total linear sample intensities remained highly uniform (mean $\left(5.65\pm 0.47\right)\times 10^{9}$, RSD=8.33%), and the ${log}_{10}$-transformed intensity distributions exhibited consistent medians (averaging 4.22±0.04, range 4.13–4.29, cross-sample RSD=0.95%) and compact interquartile boxes (IQR=1.20±0.03, range 1.14–1.25) across all QC (QC1–QC5) and experimental runs, demonstrating stable instrument sensitivity without significant signal drift. Furthermore, to evaluate full-workflow analytical reproducibility (encompassing independent sample weighing, extraction, and LC-MS acquisition), we examined the feature-wise RSD distribution (Figure S2b) and pairwise Pearson correlations across the independent composite analytical replicate triads (−1, −2, and −3) within each cultivar: across all 8283 features, the median analytical replicate RSD was 15.52% (interquartile range: 9.66–24.95%), with 82.98% (6873/8283, i.e., 83.0%) of features exhibiting a median replicate RSD<30% and 64.23% exhibiting RSD<20% (Figure S2b), while pairwise replicate Pearson correlation coefficients reached a median of r=0.992 (interquartile range: 0.976–0.996) on normalized intensities and r=0.982 (range: 0.955–0.987) on ${log}_{10}$-transformed complete features, providing a robust data foundation for subsequent statistical analyses.

### 3.4. Statistical Analysis

Prior to statistical analyses, the raw LC-MS data underwent rigorous preprocessing in Progenesis QI v2.3. First, blank filtering was performed by comparing study and QC runs against procedural extraction blanks; background artefact ions originating from extraction solvents, tubes, or mobile phases (defined as features whose mean intensity in blank runs exceeded 10% of the mean intensity in study/QC samples, i.e., sample-to-blank ratio < 10), as well as the exogenous internal standard peak itself (L-2-chlorophenylalanine), were excluded. Following the standard group-presence rule, ion peaks with missing values exceeding 50% in both experimental groups (i.e., retained only if detected in ≥50% of samples in at least one regional group) or with a relative standard deviation (RSD) > 30% across QC samples were removed. Across the retained 8283 annotated features (223,641 data points across 27 composite samples), the overall missing-value rate was 3.79% (8482 missing cells), with 6809 features (82.20%) exhibiting complete detection (0% missingness) across all 27 samples. In the primary software export, remaining zero/missing values were imputed using half of the global minimum positive intensity detected across the dataset ($2.50\times 10^{-5}$); to evaluate whether extreme fold changes were driven by this global constant imputation, a sensitivity analysis was additionally conducted using feature-wise half-minimum imputation (replacing missing values in each metabolite with half of that specific metabolite’s own minimum detected positive intensity). To correct for extraction recovery, injection volume, and within-batch ionization variations, raw peak intensities were calibrated against the spiked internal standard (L-2-chlorophenylalanine, 0.06 mg/mL) and normalized using Progenesis QI reference-run total spectral peak area scaling (yielding a uniform sample total intensity RSD of 8.33%). For putative metabolite annotation in Progenesis QI v2.3, candidate assignments were evaluated across three experimental mass spectrometric dimensions: (i) MS1 precursor mass accuracy ($S_{\mathrm{mass}}$, scored 0–100 with a mass tolerance within ±10 ppm), (ii) isotopic distribution similarity ($S_{\mathrm{isotope}}$, scored 0–100), and (iii) MS/MS fragmentation spectrum matching ($S_{\mathrm{fragmentation}}$, scored 0–100 against HMDB, LIPID MAPS v2.3, METLIN, and in-house spectral libraries). Because each dimension is weighted equally at 20% (up to 20 points each, without external retention-time or ion-mobility CCS libraries), the overall confidence score has a theoretical maximum of 60 ($\mathrm{Score}=0.20\times S_{\mathrm{mass}}+0.20\times S_{\mathrm{isotope}}+0.20\times S_{\mathrm{fragmentation}}$). Features with an overall confidence score below 36 (out of 60) were strictly excluded. To define the analytical evidence required for structural assignment in accordance with Metabolomics Standards Initiative (MSI) guidelines, retained features were stratified into two tiers: (1) MSI Level 2 putative structural annotations, which required experimental MS/MS fragmentation matching (Fragmentation Score > 0), with primary biological biomarkers requiring high-quality MS/MS concordance (Fragmentation Score > 50/100, overall Score > 48/60) and phytochemical plausibility in Glycine max; and (2) MSI Level 3 tentative formula/class annotations (Fragmentation Score = 0, Score ≥ 36/60 based on >90% MS1 mass and isotope concordance), which were treated strictly as elemental formula or class-level matches due to potential isobaric ambiguity. Because authentic chemical standards were not co-injected during the untargeted profiling, no features were claimed as MSI Level 1 confirmed identifications. The filtered and normalized data from both positive and negative ion modes were subsequently merged into a unified data matrix (8283 features across 27 composite samples).

Prior to multivariate modeling, specific data transformation and scaling procedures were applied according to model structure. Multivariate statistical modeling was performed using specific R packages: Principal component analysis (PCA) was conducted using the stats package (version 3.5.1), while partial least squares discriminant analysis (PLS-DA) and orthogonal PLS-DA (OPLS-DA) were performed using the ropls (version 1.14.1) and MetaboAnalystR (version 1.0.1) packages, respectively. Univariate statistical tests, Benjamini–Hochberg FDR adjustments, and data visualizations (including volcano plots and hierarchical clustering heatmaps) were executed using Python version 3.12. PCA was initially performed on the unit-variance (UV) scaled (mean-centered and divided by the standard deviation) data matrix to evaluate the natural distribution of the samples and the clustering of subsamples within each cultivar–location unit. For supervised modeling, normalized metabolite intensities were first ${log}_{10}$-transformed to reduce right-skewness and stabilize variance. Subsequently, PLS-DA (Pareto-scaled: mean-centered and divided by the square root of the standard deviation) and OPLS-DA (UV-scaled) were employed to characterize group separation between the sampled HLJ and LN cohorts and identify discriminative metabolic features. Regarding the number of latent components retained and model selection criteria, two principal/latent components (p1 and p2) were retained for PCA and PLS-DA, and one predictive component (p1) plus one orthogonal component (o1) were retained for OPLS-DA (Figure S3). Model complexity was selected based on the cross-validated predictive gain criterion ($\Delta Q^{2}>0.05$; where adding the second component increased cumulative 7-fold $Q^{2}$ from 0.795 to 0.949 in PLS-DA and from 0.896 to 0.962 in OPLS-DA) combined with the parsimony principle to prevent overfitting given the nine cultivar–location groups (N=9). Model fit and apparent predictive ability were initially evaluated using cumulative explained predictor variance ($R^{2}X\left(\mathrm{cum}\right)$), explained response variance ($R^{2}Y$), and cross-validated predictive ability ($Q^{2}$) via standard 7-fold cross-validation and 200-permutation testing across individual subsamples. To prevent within-cultivar data leakage caused by subsamples from the same cultivar appearing in both training and validation sets, we further validated the PLS-DA and OPLS-DA models using a group-aware cross-validation strategy (9-fold leave-cultivar-group-out cross-validation), in which all three replicates of each cultivar–location group were held out together in each validation fold. Crucially, while initial quality filtering and half-minimum imputation were performed globally prior to modeling, all fold-dependent preprocessing parameters (mean-centering and UV/Pareto scaling factors) and latent component weights were calculated independently within each cross-validation training set and subsequently applied to the held-out validation fold, with the number of retained components fixed at two (p1 + p2 for PLS-DA; p1 + o1 for OPLS-DA). Correspondingly, an exact group-restricted permutation test was conducted across all 126 ($C_{9}^{4}=126$) possible whole-cultivar group assignments to evaluate model significance while preserving the internal clustering structure.

Student’s *t*-tests (with Benjamini–Hochberg FDR correction) and fold change (FC) analyses were initially conducted across the subsample replicates to screen candidate metabolic variations. Specifically, FC was calculated on the normalized (untransformed) peak intensities as the ratio of the arithmetic mean abundance in the HLJ group to that in the LN reference group ($\mathrm{FC}={\mathrm{Mean}}_{\mathrm{HLJ}}/{\mathrm{Mean}}_{\mathrm{LN}}$), such that FC>2.0 ($\log_{2}\mathrm{FC}>1.0$) denotes higher abundance in HLJ and FC<0.5 ($\log_{2}\mathrm{FC}<-1.0$) denotes lower abundance in HLJ (i.e., higher abundance in LN) relative to the reference group. Because global half-minimum imputation ($2.50\times 10^{-5}$) can artificially inflate fold-change ratios for region-specific features that are completely undetected (100% missing) in one group, per-group missing counts (Missing_Count_HLJ and Missing_Count_LN) and sensitivity-checked fold changes under feature-wise half-minimum imputation were compiled side-by-side in Supplementary Table S1, and only metabolites unaffected by imputation artifacts were prioritized for biological interpretation. Metabolites meeting the combined criteria of Variable Importance in Projection (VIP) > 1.0 (from the OPLS-DA model), FDR < 0.05, and fold change > 2.0 or <0.5 were defined as candidate differential metabolites. Crucially, because subsamples from the same harvested seed material are clustered within cultivar and location, treating all individual observations as fully independent leads to pseudoreplication and underestimates statistical uncertainty. To account for this hierarchical dependence in the statistical analysis, we further performed a two-stage cultivar-level aggregation analysis by averaging the nested subsamples within each cultivar–location unit and evaluating the nine cultivar–location means (n=4 for HLJ vs. n=5 for LN) as the independent experimental units. Only metabolic pathways and key biomarkers showing consistent directional divergence at the cultivar–location unit level were emphasized in the biological interpretation. Finally, pathway over-representation analysis (ORA) was performed by mapping the annotated metabolites to the Kyoto Encyclopedia of Genes and Genomes (KEGG) Compound database and KEGG Orthology (ko) reference pathway maps. Specifically, all detected and putatively annotated metabolites in our dataset with valid KEGG pathway annotations (M=794 metabolites out of the 8283 annotated features) were defined as the study-specific background metabolite universe, against which the N=81 KEGG-mapped differential metabolites (out of the 1485 candidate differential features) were tested across 61 mapped pathways using a one-sided hypergeometric test. Raw nominal p-values were adjusted for multiple testing across the 61 pathways using the Benjamini–Hochberg (BH) False Discovery Rate (FDR) procedure to obtain adjusted q-values. An FDR-adjusted threshold of q<0.05 was defined for confirmatory pathway significance; because no pathway survived q<0.05, an unadjusted threshold of nominal p<0.05 (and 0.05≤p<0.10 for marginal trends) was adopted solely for exploratory, hypothesis-generating pathway prioritization, focusing on canonical plant metabolic pathways relevant to Glycine max.

## 4. Conclusions

This study systematically investigated the mature seed metabolomes of nine commercial soybean cultivars sampled from two major production regions in Northeast China, Heilongjiang (HLJ) and Liaoning (LN). Through an LC-MS-based non-targeted metabolomics approach combined with multivariate and univariate statistical modeling, we screened 1485 putatively annotated (MSI Level 2/3) differential metabolites that clearly segregate the sampled HLJ and LN cultivar–location cohorts. While these results demonstrate strong metabolic differentiation within the studied sample set, any potential application toward geographic authentication must be qualified as preliminary, as the current findings reflect candidate metabolic markers derived from single seed lots, single sites, and a single harvest season.

Our quantitative steady-state metabolite abundance data, combined with exploratory KEGG pathway mapping, reveal distinct regional metabolic accumulation patterns. Specifically, sampled Liaoning (LN) cultivars exhibit higher steady-state abundances of individual isoflavones, flavonoids, and complex secondary glycosides (driving nominal enrichment in the isoflavonoid biosynthesis pathway, p=0.0043, q=0.261). While static abundance profiles and macroclimatic comparisons (in the absence of direct site-level environmental measurements) cannot directly prove dynamic carbon resource reallocation or climatic causality, we cautiously hypothesize that these elevated flavonoid pools may be associated with secondary antioxidant capacity and local cultivar characteristics in warmer regions. Conversely, soybeans sampled from Heilongjiang (HLJ) exhibit significantly higher steady-state abundances of specific unsaturated fatty acyls, sphingolipid metabolites, and tryptophan-derived indoleamines (with tryptophan metabolism showing nominal pathway enrichment at p=0.0093, q=0.283). Based on existing cold-tolerance literature, we postulate strictly as a working hypothesis—rather than a directly measured environmental or membrane-fluidity mechanism—that these steady-state lipid and indoleamine differences may be linked to northern cultivar genetics and/or high-latitude habitat adaptation.

Collectively, these distinct metabolic signatures provide an empirical foundation of preliminary candidate markers for characterizing regional soybean germplasm in Northeast China. Crucially, as different region-specific cultivars were collected from single locations and single seed lots in each province without direct meteorological or edaphic measurements, the current observational experiment supports robust metabolic differences among the sampled soybean cultivar–location combinations, but it fundamentally cannot establish the separate variance contributions of genotype and environment, nor does it demonstrate genotype × environment (G×E) interactions. Furthermore, before any candidate metabolites identified here can be considered validated biomarkers for geographic authentication, independent external validation across additional harvest years, multiple geographic sites, independent seed lots, and broader cultivar panels is essential. Finally, because untargeted metabolomics measures static steady-state metabolite abundance rather than dynamic metabolic flux, all proposed associations between climatic conditions and specific physiological functions—such as isoflavone-associated thermal protection and lipid-associated cold acclimation—remain tentative hypotheses inferred from steady-state seed profiling. Future multi-year, multi-location trials evaluating shared genotypes across diverse environments with quantitative environmental monitoring, combined with authentic standard verification, stable-isotope metabolic flux analysis, transcriptomics, and targeted biophysical/physiological validations, will be required to decouple genetic and environmental contributions, validate robust geographic biomarkers, and advance the precision breeding of high-value, climate-resilient soybean cultivars.

## Data Availability

The completely processed feature intensity matrices, detailed metabolite annotations (including MSI confidence levels and MS/MS fragmentation scores), and statistical results supporting the conclusions of this article are transparently provided within the article and its Supplementary Materials (Tables S1–S4). The raw LC-MS instrumental data files are securely archived and will be made available by the corresponding author upon reasonable request.

## Supplementary Materials

The following supporting information can be downloaded at https://www.mdpi.com/article/10.3390/ijms27198925/s1.
