# Supplementary material for: Untargeted Metabolomics Reveals Regional and Cultivar-Associated Metabolomic Variation in Soybeans Across Northeast China
Source: Int J Mol Sci. 2026 Oct 8;27(19):8925. doi: 10.3390/ijms27198925 (PMC13665042; doi:10.3390/ijms27198925)
Supplement: Supplementary file 1 [file ijms-27-08925-s001.zip › Figure S1.pdf]

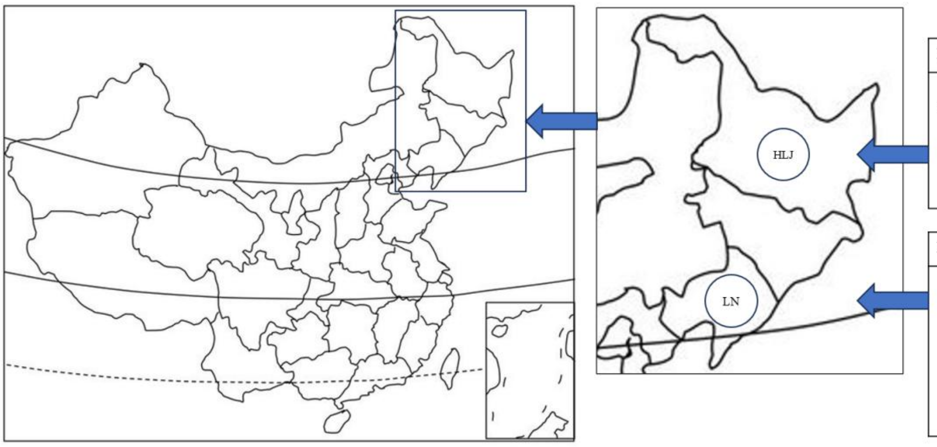

Province	Name
Hei Long Jiang (HLJ)	XN20
	HH43
	DS22
	SN52

Province	Name
Liao Ning (LN)	LD15
	LD36
	TD53
	TD67
	TF31

Figure S1. Geographical Distribution of Soybean Samples
