# Supplementary material for: Untargeted Metabolomics Reveals Regional and Cultivar-Associated Metabolomic Variation in Soybeans Across Northeast China
Source: Int J Mol Sci. 2026 Oct 8;27(19):8925. doi: 10.3390/ijms27198925 (PMC13665042; doi:10.3390/ijms27198925)
Supplement: Supplementary file 1 [file ijms-27-08925-s001.zip › Figure S2.pdf]

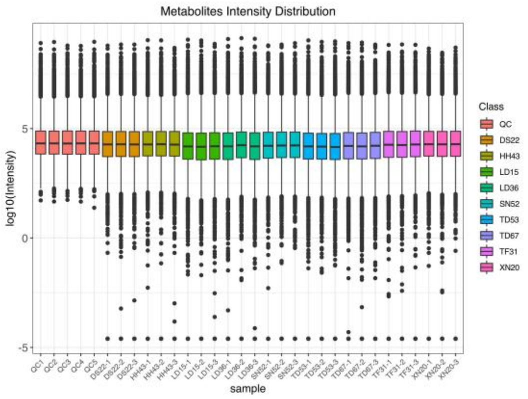

(a)

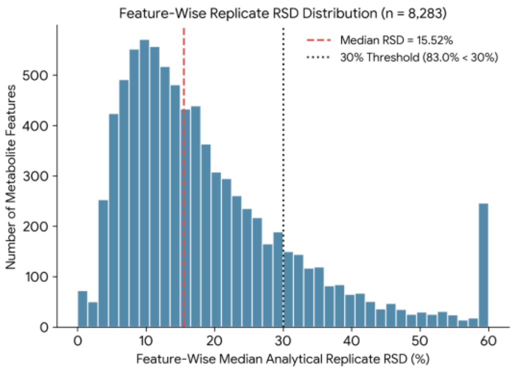

(b)

Figure S2. Data quality assessment and analytical reproducibility across quality control (QC) and experimental soybean samples. **(a)** Box plot of $\log_{10}$-transformed metabolite peak intensity distributions across periodic pooled QC samples (QC1–QC5) and the 27 composite soybean samples from nine cultivar–location groups, demonstrating consistent median intensities (4.22 ± 0.04) and compact interquartile ranges (1.20 ± 0.03) without temporal signal drift. **(b)** Feature-wise median analytical replicate relative standard deviation (RSD) distribution across all 8,283 retained metabolite features (median $\text{RSD} = 15.52\%$, with 83.0% of features exhibiting $\text{RSD} < 30\%$).
