# Supplementary material for: Untargeted Metabolomics Reveals Regional and Cultivar-Associated Metabolomic Variation in Soybeans Across Northeast China
Source: Int J Mol Sci. 2026 Oct 8;27(19):8925. doi: 10.3390/ijms27198925 (PMC13665042; doi:10.3390/ijms27198925)
Supplement: Supplementary file 1 [file ijms-27-08925-s001.zip › Figure S3.pdf]

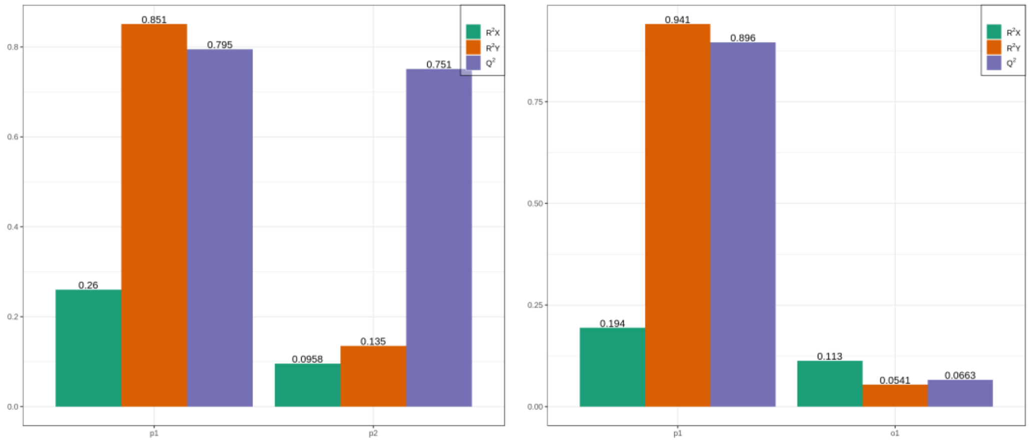

(a) (b)

Figure S3. Component-wise goodness-of-fit (R^2X and R^2Y) and predictive ability (Q^2) bar plots for the supervised multivariate models of soybean samples from Heilongjiang and Liaoning provinces. **(a)** Component-wise parameters for the PLS-DA model across the first (p1: $R^2X = 0.260$, $R^2Y = 0.851$, $Q^2 = 0.795$) and second (p2: $R^2X = 0.0958$, $R^2Y = 0.135$, $Q^2 = 0.751$) latent components. **(b)** Component-wise parameters for the OPLS-DA model across the predictive component (p1: $R^2X = 0.194$, $R^2Y = 0.941$, $Q^2 = 0.896$) and the first orthogonal component (o1: $R^2X = 0.113$, $R^2Y = 0.0541$, $Q^2 = 0.0663$).
