# Supplementary material for: Untargeted Metabolomics Reveals Regional and Cultivar-Associated Metabolomic Variation in Soybeans Across Northeast China
Source: Int J Mol Sci. 2026 Oct 8;27(19):8925. doi: 10.3390/ijms27198925 (PMC13665042; doi:10.3390/ijms27198925)
Supplement: Supplementary file 1 [file ijms-27-08925-s001.zip › Table S3.pdf]

Table S3. Detailed Information of Soybean Samples

NO.	CV.	Name	Province	Tavg	Tmax	Tmin	°E	°N	Number
1	XingNong20	XN20	HLJ	-1~12℃	35℃	-36℃	126.51	48.35	12
2	HeiHei43	HH43	HLJ	-1~12℃	38℃	-40℃	125.25	49.05	12
3	DongSheng22	DS22	HLJ	-0~13℃	35℃	-36℃	126.97	47.46	12
4	SuiNong52	SN52	HLJ	2~14℃	36℃	-34℃	127.28	46.27	12
5	LiaoDou15	LD15	LN	8~20℃	36℃	-21℃	123.12	41.53	12
6	LiaoDou36	LD36	LN	9~20℃	37℃	-21℃	120.35	40.10	12
7	TieDou53	TD53	LN	9~20℃	37℃	-20℃	121.39	41.08	12
8	TieDou67	TD67	LN	9~19℃	34℃	-18℃	122.90	39.60	12
9	TieFeng31	TF31	LN	10~20℃	34℃	-17℃	122.14	39.40	12
