# Supplementary material for: Untargeted Metabolomics Reveals Regional and Cultivar-Associated Metabolomic Variation in Soybeans Across Northeast China
Source: Int J Mol Sci. 2026 Oct 8;27(19):8925. doi: 10.3390/ijms27198925 (PMC13665042; doi:10.3390/ijms27198925)
Supplement: Supplementary file 1 [file ijms-27-08925-s001.zip › Table S4.pdf]

Table S4 LC-MS Analysis Conditions

Parameter	Condition
Chromatographic Column	ACQUITY UPLC HSS T3, 100 mm × 2.1 mm, 1.8 μm
Column Temperature	40°C
Mobile Phase A	Water containing 0.1% formic acid
Mobile Phase B	Acetonitrile containing 0.1% formic acid
Flow Rate	0.35 mL/min
Injection Volume	5 μL
Gradient Program	Initial: 5% B (1 min), Ascending: 5% to 100% B (1–12 min), Equilibration: 100% B (12–14 min), Descending: 100% to 5% B (14.2 min), Equilibrate 5% B (14.2–16 min)
Mass Spectrometry Source	Electrospray Ionization (ESI)
Mass Spectrometry Mode	Positive and Negative Ion Scanning Modes
Primary Ion Scan Mass Range	m/z 100–1200, Resolution: 70,000
Secondary Fragmentation Ion Resolution	17,500
Collision Energy (NCE)	10, 20, 40
Spray Voltage	+3.80 kV (positive ions), -3.20 kV (negative ions)
Capillary Temperature	320°C
Auxiliary Gas Heater Temperature	350°C
Sheath Gas Flow Rate	40 Arb
Auxiliary Gas Flow Rate	15 Arb
S-lens RF Level	55
